# Surface and thermal properties of synthesized cationic poly(ethylene oxide) gemini surfactants: the role of the spacer†

**DOI:** 10.1039/c9ra06577f

**Published:** 2019-09-24

**Authors:** S. M. Shakil Hussain, Muhammad Shahzad Kamal, Theis Solling, Mobeen Murtaza, Lionel Talley Fogang

**Affiliations:** Center for Integrative Petroleum Research, King Fahd University of Petroleum & Minerals Dhahran 31261 Saudi Arabia shahzadmalik@kfupm.edu.sa +966 13 860 3989 +966 13 860 8513; College of Petroleum Engineering, King Fahd University of Petroleum & Minerals Dhahran 31261 Saudi Arabia

## Abstract

The solubility and heat stability of surfactants are the prerequisites for their oilfield applications. Most commercial surfactants undergo hydrolysis at high temperature and prolonged heating at 40 °C or above leads to decomposition. In this report, three cationic poly(ethylene oxide) gemini surfactants (GSs) containing flexible and rigid spacers were synthesized for oilfield applications. The chemical structures of the GSs were elucidated with the aid of ^13^C NMR, ^1^H NMR, FT-IR, and MALDI-TOF MS. The GSs exhibit pronounced solubility in deionized water, seawater, and formation brine and no cloudiness, phase separation, or precipitation were detected after keeping GS solutions in an oven at 90 °C for three weeks. According to thermal gravimetric analysis, the degradation temperature of all the GSs was above 240 °C, which is higher than the existing oilfield temperature (≥90 °C). The critical micelle concentration (CMC) of the synthesized GSs decreases upon increasing the temperature. Additionally, CMC values were observed to increase even further with increasing salinity. The low CMC values of gemini surfactants containing a flexible structure indicate that they create a more closely packed micelle structure compared with gemini surfactants with a rigid structure. The distinct surface and thermal features of the synthesized GSs reveal them to be appropriate materials for high salinity and elevated temperature reservoirs.

## Abstract 2

Synthesis of new cationic poly(ethylene oxide) gemini surfactants containing flexible and rigid spacer groups to tolerate harsh reservoir condition.

## Graphical abstract

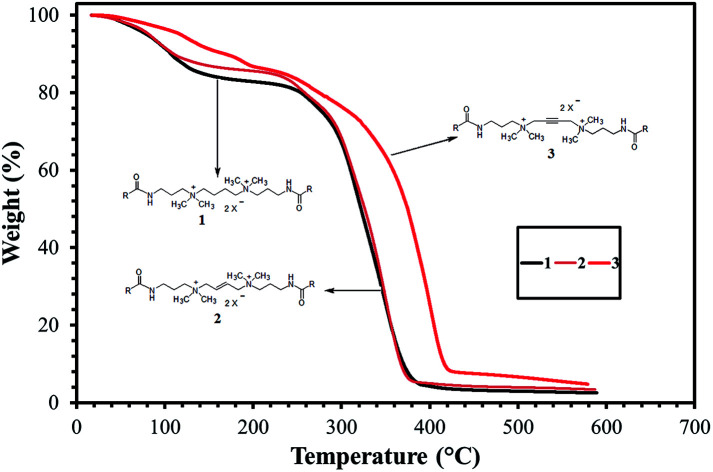

## 1. Introduction

Surfactants (surface active agents) are compounds that contain both hydrophilic and hydrophobic parts in their chemical structure.^1^ The main purpose of using surfactants for oilfield applications is to decrease interfacial tension (IFT) between brine and crude oil, to promote wettability alteration of oilfield rocks, to improve oil mobilization, and to help to form an oil bank.^2^ Surfactants are continuously applied in a range of oilfield applications including water shutoff, well completion, workovers, corrosion resistance, stimulation, drilling mud, refining, and enhanced oil recovery.^3–5^ Surfactants act as dispersants, emulsifiers, IFT reducers, wettability changers, demulsifiers, and wetting and foaming agents.^6–8^ However, many surfactants suffer in a harsh reservoir environment where high temperature and high salinity (HTHS) results in degradation. Moreover, adsorption on formation rocks makes many surfactants inapplicable.^9^

Gemini surfactants constitute a class of compounds that contain two lipophilic tail and two hydrophilic head groups chemically bonded through a spacer.^10^ Unlike monomeric surfactants, gemini surfactants reveal excellent physicochemical properties namely higher interfacial/surface activities, lower critical micelle concentration (CMC), high heat stability, high solubility in brine and water, and the ability to form unique aggregation morphologies.^11^ Due to these properties, gemini surfactants are widely used in coatings, paints, pharmaceuticals, nanomaterials, household, cosmetics, detergents, and various oilfield applications including enhanced oil recovery.^12^

Among the various classes of surfactants, the amido-amine type gemini cationic surfactants continue to receive the attention of researchers because of their high performance in industrial applications.^13^ With proper structural design of gemini cationic surfactants, it is possible to achieve a high degree of control on their physicochemical properties. Increasing the length of the lipophilic tail effectively reduces the water solubility. However, introducing ethoxy (EO) units into gemini cationic surfactants leads to enhanced water solubility.^14,15^ The presence of an amide group makes the gemini cationic surfactants biodegradable and environmentally friendly.^13^ It has been proven both theoretically and experimentally that the nature of the spacer plays a key role in the physicochemical behavior of gemini surfactants.^16–18^ The spacer could be flexible (methylene units),^19^ rigid (double bond or triple bond, benzene ring),^20^ hydrophilic (ether linkage),^21^ and hydrophobic (hydrocarbon chain).^11^ The spacer, being a critical part of a gemini surfactant, regulates adsorption on the interface layer and controls aggregation.

Herein, we report the synthesis of three new amido-amine types cationic poly(ethylene oxide) gemini surfactants (GS1–3) containing flexible and rigid spacers. The GS1 has a completely saturated and flexible butyl spacer, the GS2 possesses a rigid ethylenic spacer, and the GS3 contains a rigid acetylenic spacer. Structure characterization tools such as proton-NMR, carbon-NMR, FT-IR and MALDI-TOF MS were used to confirm the structure of GSs. The focus is to identify the effect of spacer flexibility and rigidity on the physicochemical performance of GSs and to establish a structure–property relationship. The solubility tests were done by dissolving GSs in deionized water (DW), seawater (SW), and formation brine (FW) and placed in an oven at reservoir temperature (90 °C) for three weeks. The heat stabilities of the gemini surfactants were measured using thermal gravimetric analysis (TGA). The bulk surface properties including CMC, surface tension at CMC (*γ*_cmc_), maximum surface access (*Γ*_max_), occupied surface area at the interface of air–water (*A*_min_) were investigated at varying conditions of temperature and salinity.

## 2. Experimental

### 2.1. Material

**Scheme 1:**
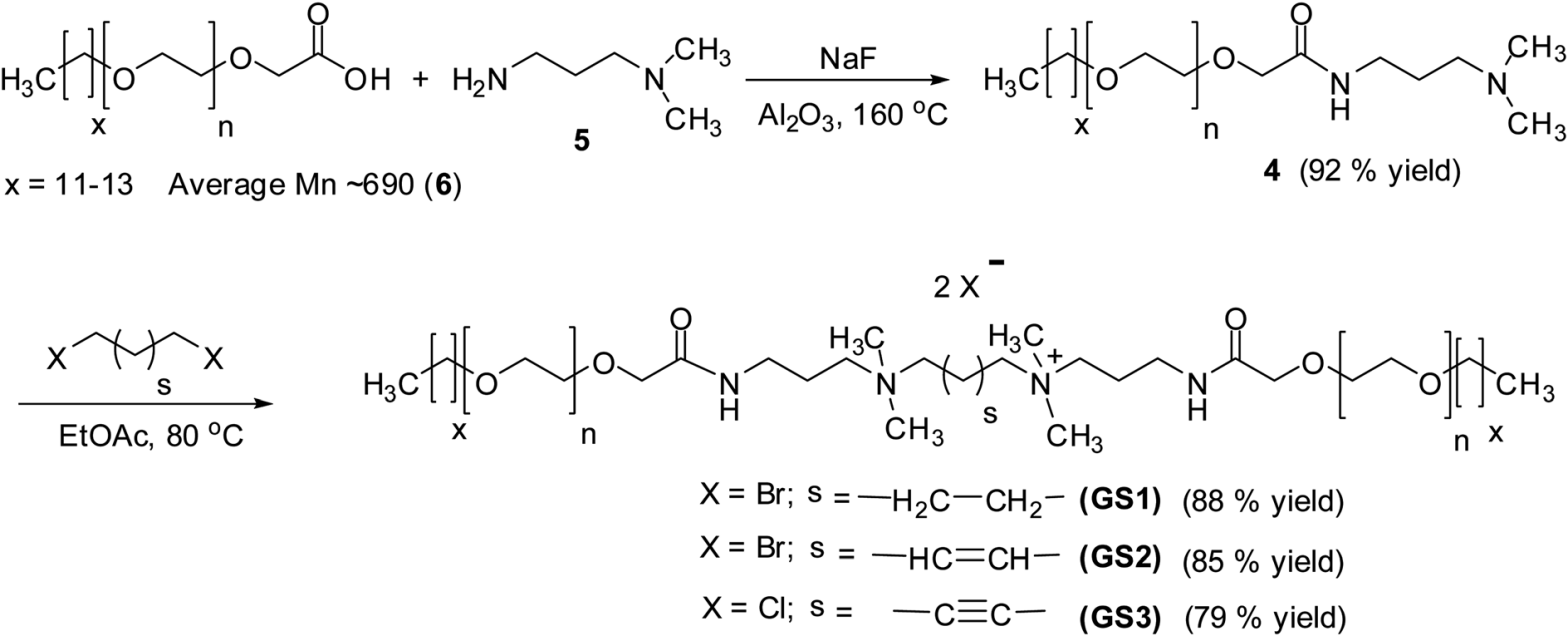
Synthesis of cationic poly(ethylene oxide) gemini surfactants GS1–3.

The cationic gemini surfactants containing EO units (GS1–3) were prepared by adopting the procedure depicted in Scheme 1.^22^ 3-(Dimethylamino)-1-propylamine (99%), glycolic acid ethoxylate lauryl ether (average *M*_n_ ∼ 690), NaF (≥99%), 1,4-dibromobutane (99%), *trans*-1,4-dibromo-2-butene (99%), 1,4-dichloro-2-butyne (99%), aluminum oxide (99.99%), were purchased from Sigma Aldrich. Seawater (SW) and formation brine (FW) were prepared by mixing CaCl_2_, Na_2_SO_4_, MgCl_2_, NaHCO_3_, NaCl. All these salts were acquired from Panreac, and the composition is provided in Table 1.

**Table 1:** The composition of salts in SW and FW

Ions	SW (g L^−1^)	FW (g L^−1^)
Na^+^	18.3	59.5
Ca^2+^	0.7	19.1
Mg^2+^	2.1	2.5
SO_4_^2−^	4.3	0.4
Cl^−^	32.2	132.1
HCO_3_^−^	0.1	0.4
Total	57.7	214

### 2.2. Structural characterization

The characterization of intermediate (4) and gemini surfactants (GS1–3) were done with the aid of FT-IR, ^1^H-NMR, ^13^C-NMR, and MALDI MS techniques. The FT-IR data was obtained using a 16F PerkinElmer FT-IR spectrometer. The NMR analysis was conducted on Jeol 1500 spectrometer and the samples were dissolved in chloroform-d tetramethylsilane as an internal standard. The MALDI mass spectrum was obtained from Bruker SolariX XR instrument in a matrix of dithranol in dichloromethane.

### 2.3. Solubility tests

Solutions of GS1–3 (10 wt%) were formed in DW, SW, FW, and placed in an oven at reservoir temperature (90 °C) for 90 days. The solubility was observed visually with the passage of time.

### 2.4. Thermal gravimetric analysis (TGA)

TGA analysis was conducted on SDT Q600 equipment from TA Instruments by heating at a rate of 20 °C min^−1^ with a constant nitrogen flow (100 mL min^−1^). The temperature interval was 30 °C to 500 °C along.

### 2.5. Surface tension measurement

The surface tension of the GSs was investigated by force tensiometer (Sigma 702, Biolin Scientific) using the Wilhelmy plate method. The measurements were performed at 30 ± 0.1 °C and 60 ± 0.1 °C. Before each measurement, the plate was rinsed with distilled water and burnt red hot on a blue flame. To check the reproducibility and proper cleanliness of the container and the ring, the surface tension of DW was determined frequently as a benchmark.

### 2.6. Synthesis

#### 2.6.1 Synthesis of intermediate (4)

The intermediate was prepared as outlined in Scheme 1. Glycolic acid ethoxylate lauryl ether (6) (average *M*_n_ ∼ 690) (30 g, 43.48 mmol) was reacted with 3-(dimethylamino)-1-propylamine (5) (8.89 g, 86.96 mmol) using sodium fluoride (0.18 g, 4.35 mmol) as a catalyst in a 250 mL round bottom flask. The reaction was left for six hours in an inert atmosphere using argon at 160 °C and the resulting water was absorbed by alumina. Subsequently, an additional 3-(dimethylamino)-1-propylamine (6.66 g, 65.22 mmol) was added to the reaction mixture which was then left for an extra four hours. Finally, the unreacted 3-(dimethylamino)-1-propylamine was evaporated by inserting a needle in the reaction flask through rubber septa and the remaining residue that contains intermediate 4 (liquid form) and sodium fluoride (solid form) was further purified by filtration technique to achieve intermediate 4.

##### 2.6.1.1 Alkyl ethoxy amidopropyl-*N*,*N*-dimethylamine (4)

Yellowish viscous material (92% yield). ^1^H-NMR (*δ* in ppm, CDCl_3_ solvent at 500 MHz): 0.88 (C*H*_3_, t, *J* = 6.7 Hz), 1.13–1.33 ((C*H*_2_)_*n*_, m), 1.54–1.64 (C*H*_2_, m), 1.69 (C*H*_2_, t, *J* = 6.9 Hz), 2.22 ((C*H*_3_)_2_, s), 2.35 (C*H*_2_, t, *J* = 7.0 Hz), 3.28–3.38 (m, C*H*_2_), 3.44 (C*H*_2_, t, *J* = 7.0 Hz), 3.57 (C*H*_2_, m), 3.61–3.69 ((–O–CH_2_–C*H*_2_–O–)_*n*_, m), 3.98 (C*H*_2_, s), 7.54 (N*H*, s).

#### 2.6.2 Synthesis of cationic poly(ethylene oxide) gemini surfactant (GS1–3)

1,4-Dibromobutan (1.12 g, 5.19 mmol) was reacted with intermediate (4) (10.0 g, 12.97 mmol) in absolute ethanol (5 mL) for 48 h (Scheme 1). Subsequently, the reaction mixture was subjected to flash column chromatography using ethanol (EtOH) as a mobile phase followed by vacuum drying to attain GS1 as a viscous material.^23^

GS2 and GS3 were in the exact same manner as GS1.

##### 2.6.2.1 Cationic poly(ethylene oxide) gemini surfactant (GS1)

Viscous material (88% yield). ^1^H NMR (*δ* in ppm, CDCl_3_ solvent at 500 MHz): 0.88 (CH_3_ × 2, t, *J* = 6.7 Hz), 1.14–1.34 ((C*H*_2_)_*n*_, m), 1.51–1.61 (C*H*_2_ × 2, m), 1.87–1.95 (CH_2_ × 2, m), 1.97–2.05 (CH_2_ × 2, m), 3.16 (CH_3_ × 4, s), 3.41–3.49 (C*H*_2_ × 2, m), 3.58–3.70 ((–O–CH_2_–CH_2_–)_*n*_, m), 4.04 (CH_2_ × 2, m), 8.11 (NH × 2, s). ^13^C NMR (*δ* in ppm, CDCl_3_ solvent at 125 MHz): 14.0, 22.6, 25.9, 29.2, 29.3, 29.5, 31.8, 35.8, 51.0, 61.9, 63.2, 69.8–70.8, 171.5. FTIR (*ν* in cm^−1^) 3416 (*ν*_N–H_), 2921 (*ν*_C–H_ asymmetric), 2860 (*ν*_C–H_ symmetric), 1652 (amide I), 1546 (amide II), 1458 (CH_2_ bend), 1350 (CH_3_ bend), 1098 (C–O–C stretching vibration), 943 (asymmetric stretch). MALDI-TOF MS *m*/*z* 768.5.

##### 2.6.2.2 Cationic poly(ethylene oxide) gemini surfactant (GS2)

Viscous material (85% yield). ^1^H NMR (*δ* in ppm, CDCl_3_ solvent at 500 MHz): 0.88 (CH_3_ × 2, t, *J* = 6.7 Hz), 1.17–1.37 ((C*H*_2_)_*n*_, m), 1.51–1.61 (C*H*_2_ × 2, m), 2.03–2.11 (CH_2_ × 2, m), 3.20 (CH_3_ × 4, s), 3.41–3.49 (C*H*_2_ × 2, m), 3.57–3.69 ((–O–CH_2_–CH_2_–)_*n*_, m), 4.03 (CH_2_ × 2, m), 6.53–6.61 (=CH × 2, m), 8.06 (NH × 2, s). ^13^C NMR (*δ* in ppm, CDCl_3_ solvent at 125 MHz): 14.0, 22.5, 26.0, 29.2, 29.4, 29.5, 31.8, 35.8, 50.9, 62.2, 64.9, 69.7–70.7, 130.1, 171.3. FTIR (*ν* in cm^−1^) 3416 (*ν*_N–H_), 2920 (*ν*_C–H_ asymmetric), 2862 (*ν*_C–H_ symmetric), 1651 (amide I), 1547 (amide II), 1460 (CH_2_ bend), 1349 (CH_3_ bend), 1097 (C–O–C stretching vibration), 940 (asymmetric stretch). MALDI-TOF MS *m*/*z* 768.5.

##### 2.6.2.3 Cationic poly(ethylene oxide) gemini surfactant (GS3)

Viscous material (79% yield). ^1^H NMR (*δ* in ppm, CDCl_3_ solvent at 500 MHz): 0.88 (CH_3_ × 2, t, *J* = 6.7 Hz), 1.16–1.36 ((C*H*_2_)_*n*_, m), 1.52–1.62 (C*H*_2_ × 2, m), 2.04–2.12 (CH_2_ × 2, m), 3.30 (CH_3_ × 4, s), 3.31–3.39 (C*H*_2_ × 2, m), 3.57–3.68 ((–O–CH_2_–CH_2_–)_*n*_, m), 4.13 (CH_2_ × 2, m), 8.00 (NH × 2, s). ^13^C NMR (*δ* in ppm, CDCl_3_ solvent at 125 MHz): 14.0, 22.6, 26.0, 29.2, 29.4, 29.5, 31.8, 35.8, 51.0, 60.6, 62.4, 69.9–70.7, 80.2, 171.4. FTIR (*ν* in cm^−1^) 3405 (*ν*_N–H_), 2921 (*ν*_C–H_ asymmetric), 2860 (*ν*_C–H_ symmetric), 1655 (amide I), 1546 (amide II), 1461 (CH_2_ bend), 1353 (CH_3_ bend), 1100 (C–O–C stretching vibration), 943 (asymmetric stretch). MALDI-TOF MS *m*/*z* 768.5.

## 3. Results and discussion

Three cationic poly(ethylene oxide) gemini surfactant (GS1–3) with flexible and rigid spacers were prepared by treating 3-(dimethylamino)-1-propylamine (5) with glycolic acid ethoxylate lauryl ether (6) using NaF as a catalyst (Scheme 1). The resulting amido-amine intermediate (4) was separately stirred with 1,4-dibromobutane, *trans*-1,4-dibromo-2-butene, and 1,4-dichloro-2-butyne to achieved GS1, GS2, and GS3, respectively.

### 3.1. Chemical structure confirmation

**Fig. 1:**
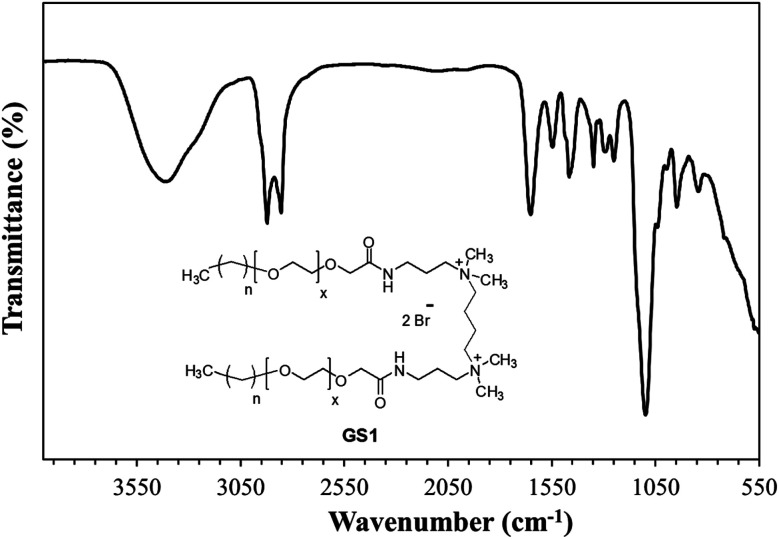
FT-IR spectrum of cationic poly(ethylene oxide) gemini surfactant (GS1).

**Fig. 2:**
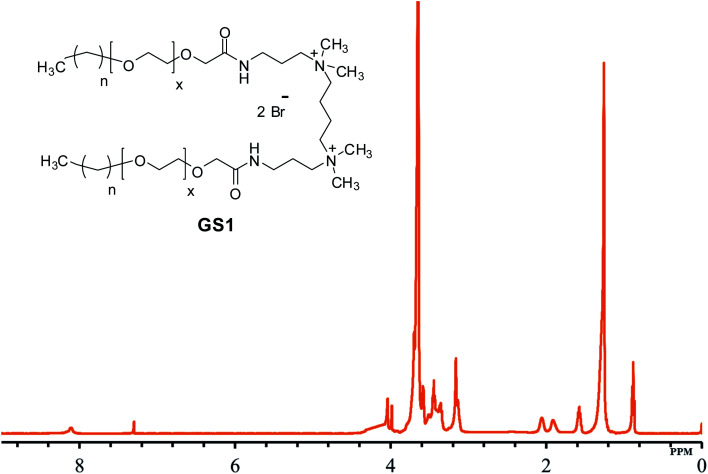
^1^H-NMR of cationic poly(ethylene oxide) gemini surfactant (GS1).

**Fig. 3:**
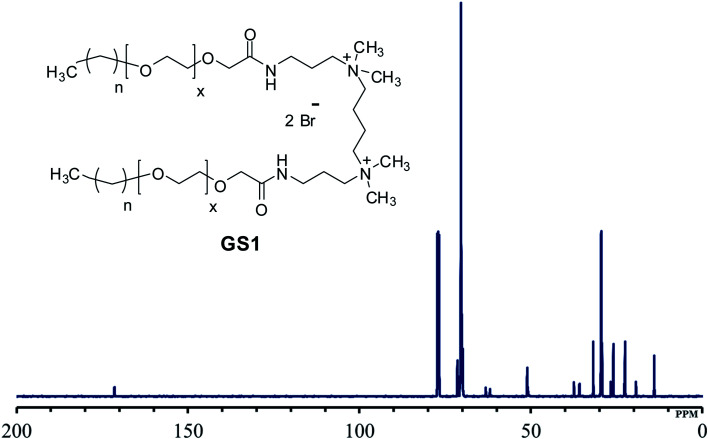
^13^C-NMR of cationic poly(ethylene oxide) gemini surfactant (GS1).

**Fig. 4:**
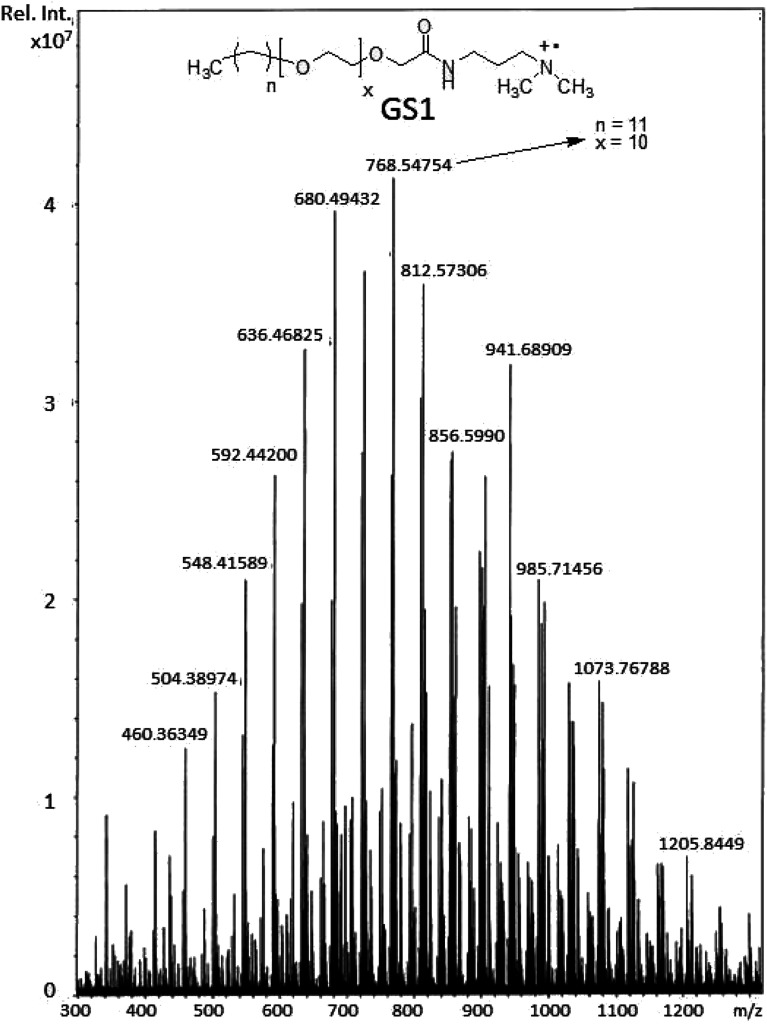
MALDI-TOF MS of cationic poly(ethylene oxide) gemini surfactant (GS1).

The structural verification is exemplified for GS1. The FT-IR spectrum of GS1 (Fig. 1) has an adsorption band at 3416 cm^−1^ which is in agreement with the stretching vibration of the N–H group. The adsorption at 2860 cm^−1^ as well as 2921 cm^−1^ can be assigned to the symmetric and asymmetric vibration of the CH_2_ group. The peak at 1652 cm^−1^ and 1546 cm^−1^ is in agreement with the presence of a C=O stretch in amide I and amide II band.^24^ The adsorption band at 1458 cm^−1^ and 1350 cm^−1^ is a result of CH_2_ and CH_3_ bending vibrations, respectively. The adsorption at 1098 cm^−1^ reflects the stretching vibration of the ether group.^25^ Detailed FT-IR spectra of synthesized gemini surfactants with characteristics peaks and their corresponding numbers are given in ESI (Fig. S1–S3).† From the ^1^H-NMR results (Fig. 2), the triplet peak at *δ* 0.88 ppm and *δ* 1.14–1.34 ppm results from the CH_3_ and CH_2_ groups [C*H*_3_–(C*H*_2_)_*n*_–] of the surfactant tail, respectively. The singlet at *δ* 3.16 ppm agrees with the presence of CH_3_ groups connected to the quaternary nitrogen [–(C*H*_3_)_2_–*N*–(CH_2_)_4_–*N*–(C*H*_3_)_2_–]. The methylene groups of the ethylene oxide chain (–O–CH_2_–C*H*_2_–O–CH_2_–CH_2_–) give rise to the multiplet signals at *δ* 3.58–3.70 ppm. The singlet at *δ* 4.04 ppm is taken to be a result of the methylene group next to the carbonyl group [–CH_2_–CH_2_–O–CH_2_–C=O–NH–]. The at the singlet at *δ* 8.11 ppm could be coupled to the amide proton [–CH_2_–C=O–N*H*–CH_2_–].^13^^1^H-NMR spectrum of the synthesized surfactants with peak integration is also provided in ESI (Fig. S4–S6).† From the ^13^C-NMR data (Fig. 3), the *C*H_3_ and *C*H_2_ groups of the hydrophobic tail are identified at *δ* 14.0 ppm and *δ* 22.6–31.8 ppm, respectively. The *C*H_3_ groups linked to the quaternary nitrogen [–(*C*H_3_)_2_–*N*–(CH_2_)_4_–*N*–(*C*H_3_)_2_–] could be associated with the signals observed at *δ* 51.0 ppm. The signals at *δ* 61.9 ppm and *δ* 63.2 ppm refer to the CH_2_ groups at the quaternary nitrogen [–CH_2_–*N*–(CH_3_)_2_–CH_2_–(CH_2_)_2_–CH_2_–(CH_3_)_2_–*N*–CH_2_–]. Methylene groups of ethylene oxide chain (–CH_2_–*C*H_2_–O–CH_2_–*C*H_2_–O–) give rise to the cluster of peaks at *δ* 69.8–70.8 ppm. The peak that appeared at *δ* 171.1 ppm is associated with the amide group [–O–CH_2_–CH_2_–*C*=O–NH–]. The mass spectrometric measurement (gave rise to the MALDI spectrum in Fig. 4) for GS1. The base peak is at *m*/*z* 768.5. At first glance, this seems to be in agreement with the principal component being GS1 where *n* = 9 and *x* = 10 because this gives a mass of 1537 for the gemini surfactant and the peak at 768.5 is then a result of the two positive charges (*m*/*z* = 1537/2). However, the mass analysis of the species with the ethylenic and acetylenic spacers proved this hypothesis wrong because in those cases the position of the base peak is unchanged at 768.5, whereas it should have been 767.5 and 766.5, respectively. The only way that this result is compatible is if the same ionic species is formed from all three compounds. This can be accomplished by homolytic cleavage of the bond between the quaternary-N and the terminal carbon of the spacer. The decomposition gives rise to the radical cation shown Fig. 4 and leads to the conclusion that GS1 with *n* = 11 and *x* = 10 is the most predominant component.

### 3.2. Solubility and salt tolerance

**Fig. 5:**
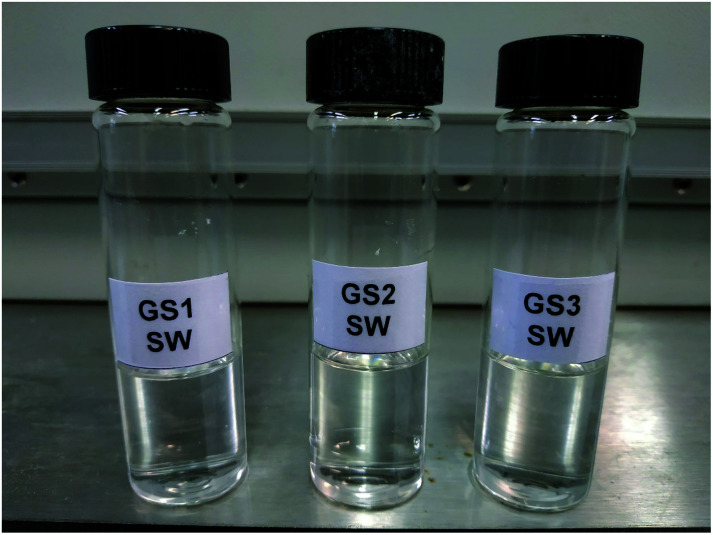
GS1–3 solutions in SW.

**Fig. 6:**
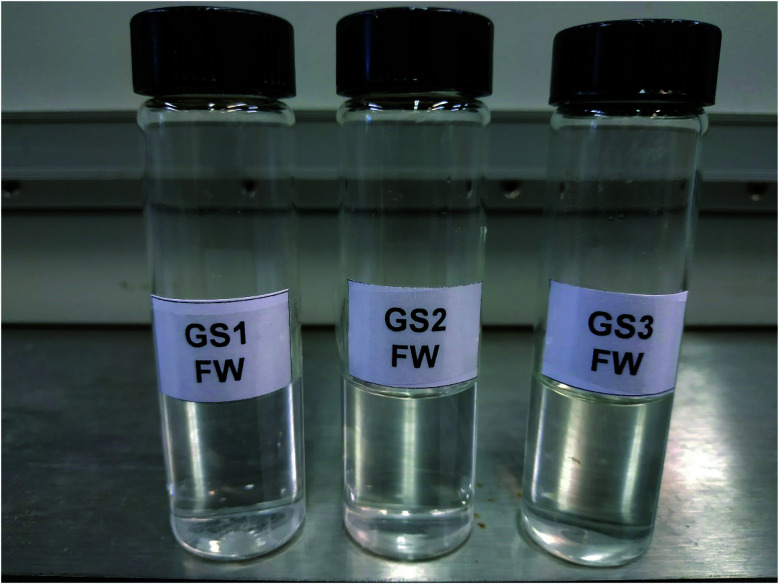
GS1–3 solutions in FW.

In order for a surfactant to be useful for oilfield applications, it must be soluble in the injection water (usually seawater) and the formation brine (FW). It has been shown that increasing the length of a surfactant tail leads to lower solubility of surfactant.^26^ However, intercalation of EO groups among hydrophilic head and lipophilic tail groups can enhance the solubility of the surfactants.^27^ The present results are indicating that the addition of specific EO units in the chemical structure of the synthesized surfactants (GS1–3) can significantly increase the solubility of the surfactants. This solubility enhancement phenomenon can be rationalized in terms of increased hydrogen bonding among ether oxygen of the EO group and aqueous molecules. Additionally, we speculate that longer ether chains may enable encapsulation of cations. An increasing number of EO units in the molecule results in a higher degree of stabilization of the surfactant molecule by hydrogen bonding between the EO groups and aqueous molecules which eventually enhance the solubility of the surfactants in all kinds of water. The surfactants were mixed into in SW and FW and kept in an oven at 90 °C (typical reservoir temperature) for three weeks. The composition of each salt in simulated SW and FW is described in Table 1 and the results of solubility are outlined in Fig. 5 and 6. Regardless of the nature of spacer groups, all three surfactants (GS1–3) showed pronounced solubility in all kinds of water at room temperature as well as at 90 °C. The solutions of GS1–3 remained transparent at 90 °C up to three weeks without any cloudiness, phase separation, or precipitation.

### 3.3. Heat tolerance

**Fig. 7:**
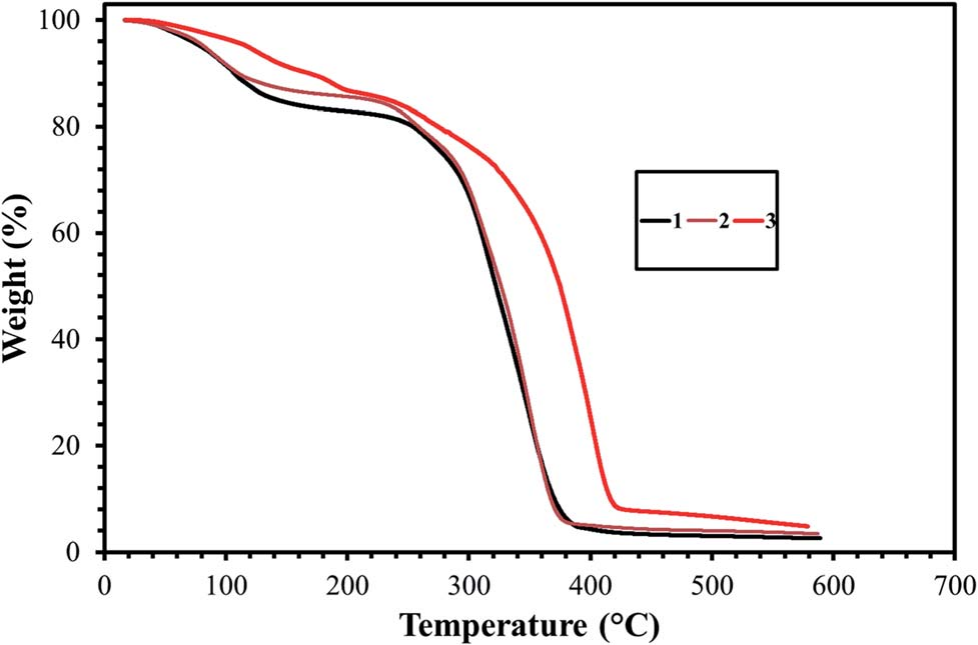
Results of thermal gravimetric analysis (TGA) of GS1–3.

Heat tolerance is one of the most important properties for any surfactant selected for an oilfield application. In this experiment, the thermal stabilities of the three surfactants were assessed with the aid of TGA. Fig. 7 displayed the TGA thermograms of the GS1–3 in the presence of nitrogen. The initial weight-loss of GS1, GS2, and GS3 was 13%, 11%, and 10%, respectively, as a result of evaporation of residual water and solvent. A sharp slope was observed at 247 °C, 249 °C, and 287 °C for GS1, GS2, and GS3, respectively, indicating the degrading effect of heat on the chemical structures of GS1–3. When comparing thermal stabilities, all three surfactants (GS1–3) have quite similar degradation behavior. In fact, there is not much difference between the thermal stabilities of GS1 and GS2. However, GS3 thermally degrades slower than GS2 and GS1. This may be attributed to the fact that the GS3 contain a triple bond in the spacer group and the electrons of the triple bond are more polarizable and produce higher London dispersion forces.^28^ In general, the range of temperatures at which thermal loss for GS1–3 was found to be greater than the existing oilfield temperature (≥90 °C) which leaves the conclusion that the synthesized surfactants (GS1–3) are capable of retaining their chemical structure at reservoir conditions.

### 3.4. Surface tension measurement

**Fig. 8:**
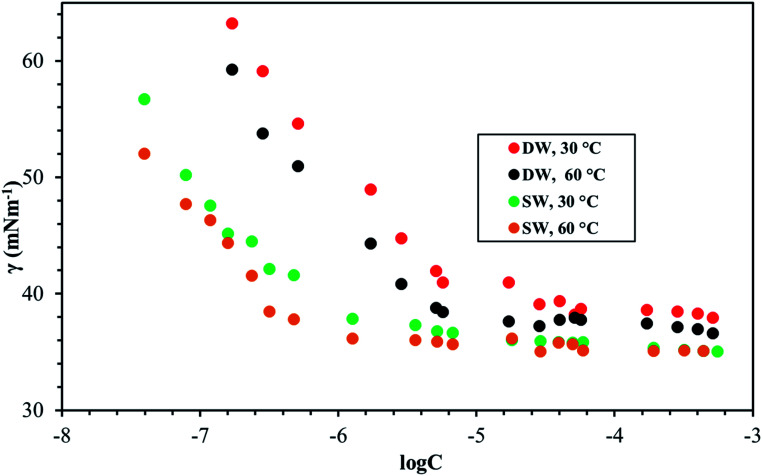
Surface tension of GS1 at various salinities and temperatures.

**Fig. 9:**
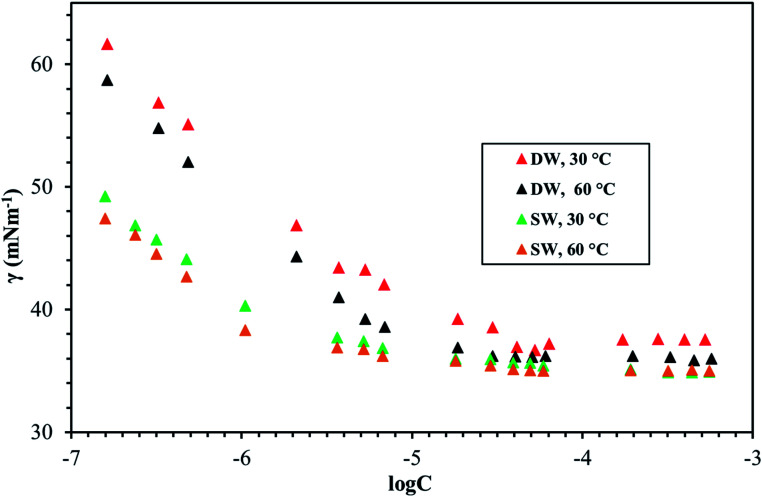
Surface tension of GS2 at various salinities and temperatures.

**Fig. 10:**
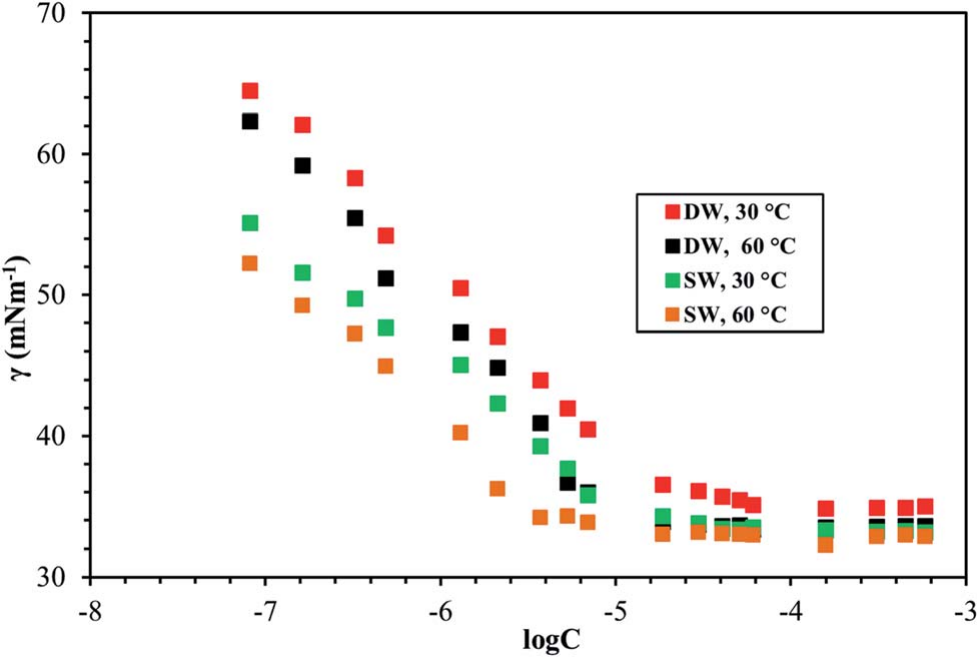
Surface tension of GS3 at various salinities and temperatures.

Fig. 8–10 shows the surface tension of GS1–3 at varying conditions of temperature and salinity. The derived surface properties are given in Table 2. The surface tension decreases continuously with increasing concentration of all the surfactants until the CMC is reached. There is negligible change in the surface tension upon extra addition of the surfactant beyond CMC. For GS1–3, the surface tension reduces with increasing temperature and salinity. At any given concentration, the surface tension of the GSs solution in the absence of salts was higher compared with the surface tension in presence of salts. Similarly, for GS1–3, the surface tension at 60 °C was lower compared to the surface tension at 30 °C. The change in surface tension with temperature and salinity for a similar class of gemini surfactants have been reported previously.^29^ Other surface properties such as CMC, surface tension corresponding to CMC (*γ*_cmc_), maximum surface access (*Γ*_max_) and minimum area per molecule (*A*_min_) were estimated from the surface tension data. The following equations were used to calculate the surface properties:^30^1
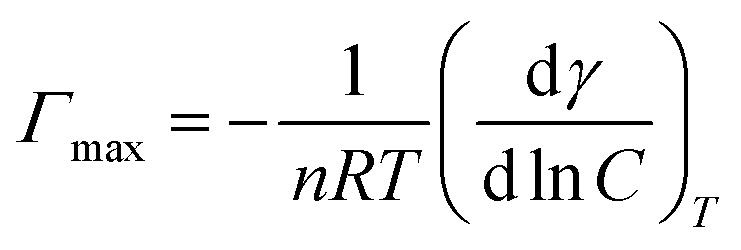

2 *A*_min_ = 10^18^/*N*_A_*Γ*_max_

Here d*γ*/d ln *C* is the slope below CMC in surface tension plot, *R* represents the gas constant, *C* is the concentration of the surfactant, *T* is the temperature, *N*_A_ Avogadro's number and *n* was taken as 3 for the gemini surfactant.

**Table 2:** Surface properties of GS1–3 at various temperatures and salinities

Surfactant	Brine	*T* (°C)	CMC (mol L^−1^)	*γ*_cmc_ (mN m^−1^)	*Γ*_max_ × 10^7^ (mol m^−2^)	*A*_min_ (nm^2^)
GS1	DW	30	8.51 × 10^−6^	38.4	8.09	2.05
GS1	DW	60	6.02 × 10^−6^	37.1	6.86	2.41
GS1	SW	30	3.54 × 10^−7^	37.4	7.98	2.08
GS1	SW	60	3.16 × 10^−7^	36.9	6.81	2.43
GS2	DW	30	1.12 × 10^−5^	39.10	6.95	2.38
GS2	DW	60	8.12 × 10^−6^	36.21	6.56	2.52
GS2	SW	30	6.77 × 10^−7^	37.66	6.08	2.73
GS2	SW	60	5.88 × 10^−7^	37.12	5.86	2.83
GS3	DW	30	1.51 × 10^−5^	35.8	6.78	2.44
GS3	DW	60	1.01 × 10^−5^	33.85	6.37	2.60
GS3	SW	30	3.76 × 10^−6^	33.5	4.72	3.51
GS4	SW	60	3.29 × 10^−6^	33.21	4.65	3.57

The CMC and surface tension corresponding to the CMC decreased after enhancing the temperature and salinity for all GS1–3. The surface tension relies on the interaction of the molecules of surfactants at the water–micelle interface. Any internal or external factors that enhance the presence of surfactant at the interface will result in the reduction of the surface tension. Addition of salts increases the presence of the surfactant at the interface due to reduced hydration of surfactant molecules. Addition of salts also results in more close packing of surfactant molecules at the interface by reducing the repulsion among the surfactant molecules. Thus, salinity increase results in a reduction of the CMC and *γ*_cmc_. GS1–3 contains the same number of hydrophobic units (tail group) and hydrophilic units (EO groups), the same headgroups (ammonium), and approximately the same length of the spacer (C4). Therefore, the nature of the spacer is the key factor in identifying the difference between aggregation morphologies. GS1 contains a flexible spacer and exhibited the lowest CMC compared with GS2 (rigid with a double bond) and GS3 (rigid with a triple bond). The CMC of GS1–3 at 30 °C and 60 °C as well as different salinities (DW and SW) was in the order of GS1 < GS2 < GS3. Such phenomena can be described by the spacer group flexibility. The flexible spacer of GS1 makes it easier to be situated at the water–micelle interface and forms a more closely packed micelle structure. However, the high CMC value of the surfactants with rigid spacers (GS2 and GS3) suggest that there may be a steric hindrance between intermolecular chain–chain association which makes them difficult to organize at the water–micelle interface.^31^ Moreover, as the polarity (or hydrophilicity) of the spacer group increases (GS1 < GS2 < GS3), the CMC also increases in agreement with previous studies.^32^ In deionized water, the minimum CMC (6.02 × 10^−6^ mol L^−1^) was observed at 60 °C for GS1 followed by GS2 and GS3. This behavior is consistent at both temperature and salinity for GS1–3. These results show that the surface properties of the GSs can be influenced by the nature of the spacer group. The gemini surfactants with a flexible spacer usually make a mixture of vesicles and micelles and the gemini surfactants having a rigid spacer usually form vesicles in aqueous solution.^33^ The CMC values of the synthesized surfactants vary from 3.16 × 10^−7^ mol L^−1^ to 1.51 × 10^−5^ mol L^−1^. The extremely low CMC values make these surfactants cost-effective for various applications such as those related to oil and gas. The reported values for a similar class of gemini surfactants but without ethoxylation range from 8.0 × 10 ^−3^ mol L^−1^ to 12.5 × 10^−3^ mol L^−1^.^24^ The surface access at the air–water interface decreases with increase in temperature and salinity. *A*_min_ increased with increasing temperature and salinity. Although the surface properties are influenced by the spacer modification, the difference itself is not significant. In general, all the synthesized GSs show extremely low CMC in deionized and saline water.

## 4. Conclusion

In conclusion, three cationic poly(ethylene oxide) gemini surfactants (GS1–3) containing the same chemical structure except nature of the spacer group were synthesized. The effect of flexible and rigid spacers on the surface and thermal properties of the GSs was investigated. The results show that the nature of the spacer has an important effect on the aggregation morphologies of the GSs. All three surfactants (GS1–3) showed pronounced solubility and salt tolerance in all kinds of water, and the solutions of GS1–3 remained transparent at 90 °C up to 3 weeks without any cloudiness, phase separation, or precipitation. TGA thermograms showed that the synthesized GSs have a higher thermal degradation temperature than the real oilfield temperature (90 °C). Moreover, GS3 exhibited higher thermal stability compared to GS2 and GS1. The decomposition temperature of the GS1–3 was on the order of GS1 (247 °C) < GS2 (249 °C) < GS3 (287 °C). It was observed that the introduction of spacer rigidity (GS2 with double or GS3 with triple bond) shifts the CMC to higher values. The flexible spacer in GS1 stimulates micelle formation and creates a more closely packed micelle structure which leads to a lower CMC. The IFT, rheology, and foam analysis is already underway in our laboratories.

## Conflicts of interest

There are no conflicts to declare.

## Supplementary Material

RA-009-C9RA06577F-s001

## References

[cit1] Alam M. S., Siddiq A. M., Natarajan D., Kiran M. S., Baskar G. (2019). Physicochemical properties and bioactivity studies of synthesized counterion coupled (COCO) gemini surfactant, 1,6-bis (N,N-hexadecyldimethylammonium) adipate. J. Mol. Liq..

[cit2] Olajire A. A. (2014). Review of ASP EOR (alkaline surfactant polymer enhanced oil recovery) technology in the petroleum industry: Prospects and challenges. Energy.

[cit3] Alzahid Y. A., Mostaghimi P., Walsh S. D., Armstrong R. T. (2019). Flow regimes during surfactant flooding: the influence of phase behaviour. Fuel.

[cit4] Sultan A. , Azad M., Hussein I. and Mahmoud M., Rheological assessment of VES as an EOR fluid in carbonate reservoir, SPE EOR conference at oil and gas West Asia, Society of Petroleum Engineers, 2014

[cit5] Azad M. S. , Sultan A. S., Nuaim S. A., Mahmoud M. and Hussein I. W., Could VES be a part of Hybrid option to recover Heavy oil in Complex Heavy oil Reservoirs, SPE Heavy Oil Conference-Canada, Society of Petroleum Engineers, 2014

[cit6] Hajibagheri F., Hashemi A., Lashkarbolooki M., Ayatollahi S. (2018). Investigating the synergic effects of chemical surfactant (SDBS) and biosurfactant produced by bacterium (Enterobacter cloacae) on IFT reduction and wettability alteration during MEOR process. J. Mol. Liq..

[cit7] Sagir M., Tan I. M., Mushtaq M., Ismail L., Nadeem M., Azam M. R., Hashmet M. R. (2014). Novel surfactant for the reduction of CO2/brine interfacial tension. J. Dispersion Sci. Technol..

[cit8] Ahmed S., Elraies K., Hashmet M., Alnarabiji M. (2018). Empirical modeling of the viscosity of supercritical carbon dioxide foam fracturing fluid under different downhole conditions. Energies.

[cit9] Abbas A. H., Sulaiman W. R. W., Jaafar M. Z., Olayink A. A., Ebrahimi S. S., Elrufai A. (2018). Numerical study for continuous surfactant flooding considering adsorption in heterogeneous reservoir. Journal of King Saud University-Engineering Sciences.

[cit10] Menger F., Littau C. (1993). Gemini surfactants: a new class of self-assembling molecules. J. Am. Chem. Soc..

[cit11] Luo W., Ouyang J., Antwi P., Wu M., Huang Z., Qin W. (2019). Microwave/ultrasound-assisted modification of montmorillonite by conventional and gemini alkyl quaternary ammonium salts for adsorption of chromate and phenol: Structure-function relationship. Sci. Total Environ..

[cit12] Brycki B. E. , Kowalczyk I. H., Szulc A., Kaczerewska O. and Pakiet M., Multifunctional Gemini Surfactants: Structure, Synthesis, Properties and Applications, Application and Characterization of Surfactants, InTech, 2017

[cit13] Wang Y., Jiang Y., Geng T., Ju H., Duan S. (2019). Synthesis, surface/interfacial properties, and biological activity of amide-based Gemini cationic surfactants with hydroxyl in the spacer group. Colloids Surf., A.

[cit14] Shakil Hussain S., Animashaun M. A., Kamal M. S., Ullah N., Hussein I. A., Sultan A. S. (2016). Synthesis, characterization and surface properties of amidosulfobetaine surfactants bearing odd-number hydrophobic tail. J. Surfactants Deterg..

[cit15] Hussain S. S., Fogang L. T., Kamal M. S. (2018). Synthesis and performance evaluation of betaine type zwitterionic surfactants containing different degrees of ethoxylation. J. Mol. Struct..

[cit16] Parikh K., Singh S., Desai A., Kumar S. (2019). An interplay between spacer nature and alkyl chain length on aqueous micellar properties of cationic Gemini surfactants: A multi-technique approach. Journal of Molecular Liquids.

[cit17] De S., Aswal V. K., Goyal P. S., Bhattacharya S. (1996). Role of spacer chain length in dimeric micellar organization. Small angle neutron scattering and fluorescence studies. J. Phys. Chem. A.

[cit18] Diamant H., Andelman D. (1995). Dimeric surfactants: a simplified model for the spacer chain. Langmuir.

[cit19] Taleb K., Pillin I., Grohens Y., Saidi-Besbes S. (2018). Gemini surfactant modified clays: Effect of surfactant loading and spacer length. Appl. Clay Sci..

[cit20] Laschewsky A., Lunkenheimer K., Rakotoaly R. H., Wattebled L. (2005). Spacer effects in dimeric cationic surfactants. Colloid Polym. Sci..

[cit21] Kaczerewska O., Brycki B., Ribosa I., Comelles F., Garcia M. T. (2018). Cationic gemini surfactants containing an O-substituted spacer and hydroxyethyl moiety in the polar heads: self-assembly, biodegradability and aquatic toxicity. J. Ind. Eng. Chem..

[cit22] Chu Z., Feng Y. (2009). A facile route towards the preparation of ultra-long-chain amidosulfobetaine surfactants. Synlett.

[cit23] Zana R., Benrraou M., Rueff R. (1991). Alkanediyl-. alpha, omega.-bis (dimethylalkylammonium bromide) surfactants. 1. Effect of the spacer chain length on the critical micelle concentration and micelle ionization degree. Langmuir.

[cit24] Ghumare A. K., Pawar B. V., Bhagwat S. S. (2013). Synthesis and antibacterial activity of novel amido-amine-based cationic gemini surfactants. J. Surfactants Deterg..

[cit25] Al-Sabagh A., Azzam E., Mahmoud S., Saleh N. (2007). Synthesis of ethoxylated alkyl sulfosuccinate surfactants and the investigation of mixed solutions. J. Surfactants Deterg..

[cit26] Kamal M. S., Shakil Hussain S. M., Fogang L. T. (2018). A Zwitterionic Surfactant Bearing Unsaturated Tail for Enhanced Oil Recovery in High-Temperature High-Salinity Reservoirs. J. Surfactants Deterg..

[cit27] Negin C., Ali S., Xie Q. (2017). Most common surfactants employed in chemical enhanced oil recovery. Petroleum.

[cit28] Menger F., Keiper J., Azov V. (2000). Gemini surfactants with acetylenic spacers. Langmuir.

[cit29] Hussain S. S., Kamal M. S. (2017). Effect of large spacer on surface activity, thermal, and rheological properties of novel amido-amine cationic gemini surfactants. J. Mol. Liq..

[cit30] Hussain S. S., Kamal M. S., Fogang L. T. (2018). Effect of internal olefin on the properties of betaine-type zwitterionic surfactants for enhanced oil recovery. J. Mol. Liq..

[cit31] Wang X., Wang J., Wang Y., Yan H., Li P., Thomas R. K. (2004). Effect of the nature of the spacer on the aggregation properties of gemini surfactants in an aqueous solution. Langmuir.

[cit32] Garcia M. T., Kaczerewska O., Ribosa I., Brycki B., Materna P., Drgas M. (2017). Hydrophilicity and flexibility of the spacer as critical parameters on the aggregation behavior of long alkyl chain cationic gemini surfactants in aqueous solution. J. Mol. Liq..

[cit33] Zhu D.-Y., Cheng F., Chen Y., Jiang S.-C. (2012). Preparation, characterization and properties of anionic gemini surfactants with long rigid or semi-rigid spacers. Colloids Surf., A.

