# Supplementary material for: Surface and thermal properties of synthesized cationic poly(ethylene oxide) gemini surfactants: the role of the spacer
Source: RSC Adv. 2019 Sep 24;9(52):30154–63. doi: 10.1039/c9ra06577f (PMC9072132; doi:10.1039/c9ra06577f)
Supplement: RA-009-C9RA06577F-s001 [file RA-009-C9RA06577F-s001.pdf]

Electronic Supplementary Material (ESI) for RSC Advances.
This journal is © The Royal Society of Chemistry 2019

Surface and thermal properties of the synthesized cationic poly(ethylene oxide) gemini surfactants: Role of spacer rigidity

S. M. Shakil Hussain[1], Muhammad Shahzad Kamal[1], Theis Solling[1], Mobeen Murtaza[2], Lionel Talley Fogang[1]

[1]Center for Integrative Petroleum Research, King Fahd University of Petroleum & Minerals, Dhahran 31261, Saudi Arabia.

2 College of Petroleum Engineering, King Fahd University of Petroleum & Minerals, Dhahran 31261, Saudi Arabia

Corresponding Author: Dr. Muhammad Shahzad Kamal

Email: shahzadmalik@kfupm.edu.sa

Telephone: +966 13 860 8513

Fax: +966 13 860 3989

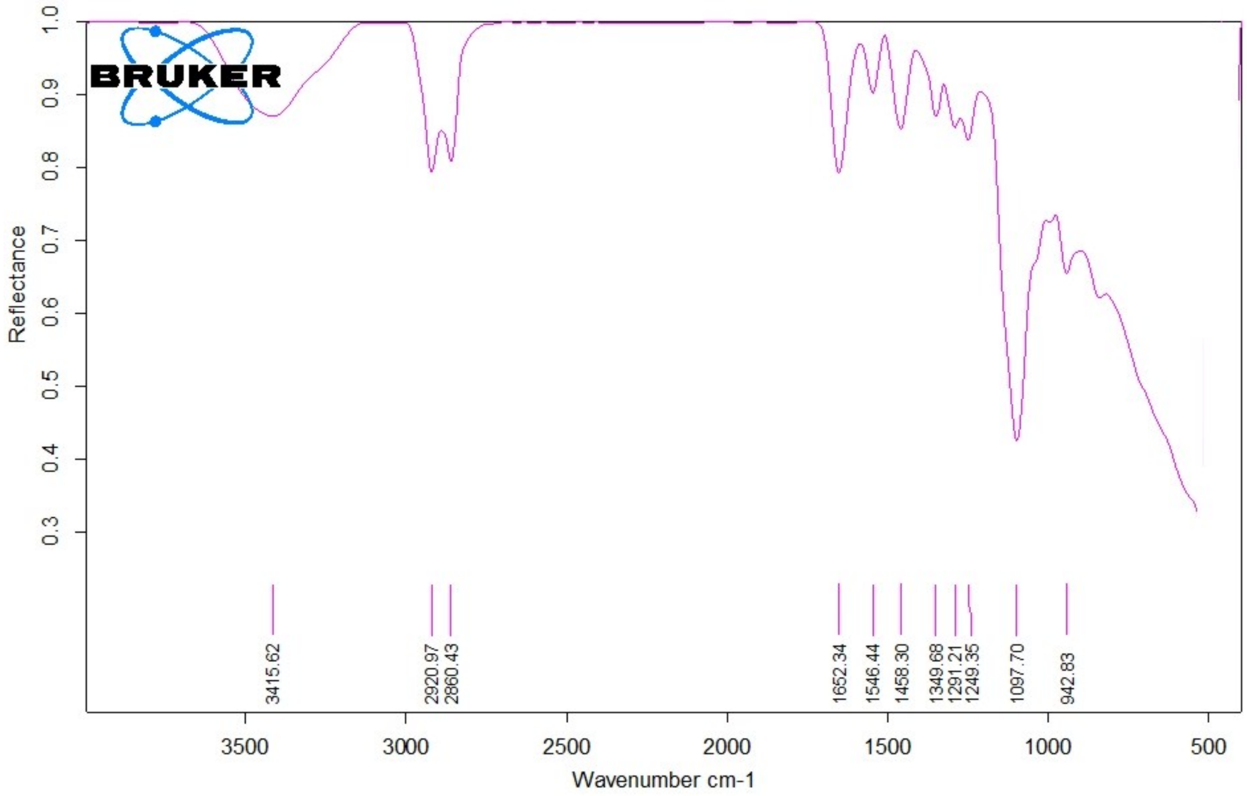

Fig. S1. FT-IR spectrum of cationic poly(ethylene oxide) gemini surfactant (**GS1**)

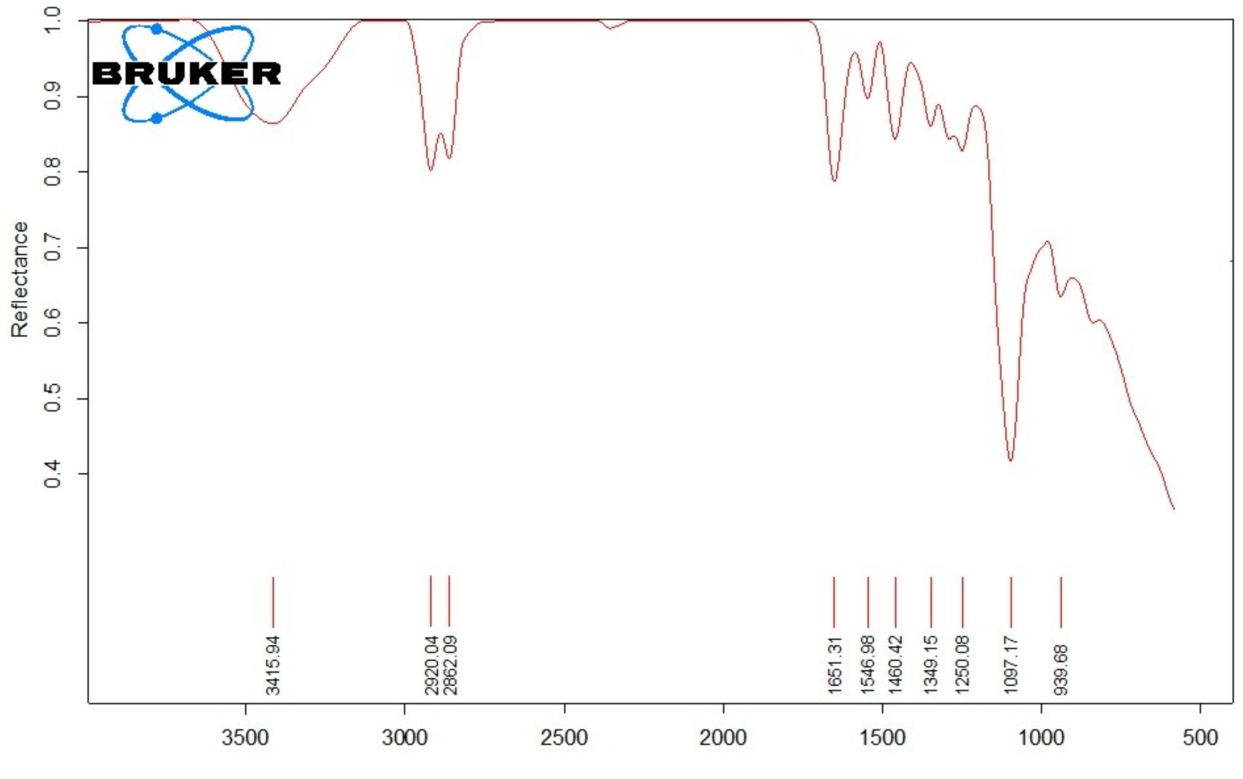

Fig. S2. FT-IR spectrum of cationic poly(ethylene oxide) gemini surfactant (**GS2**)

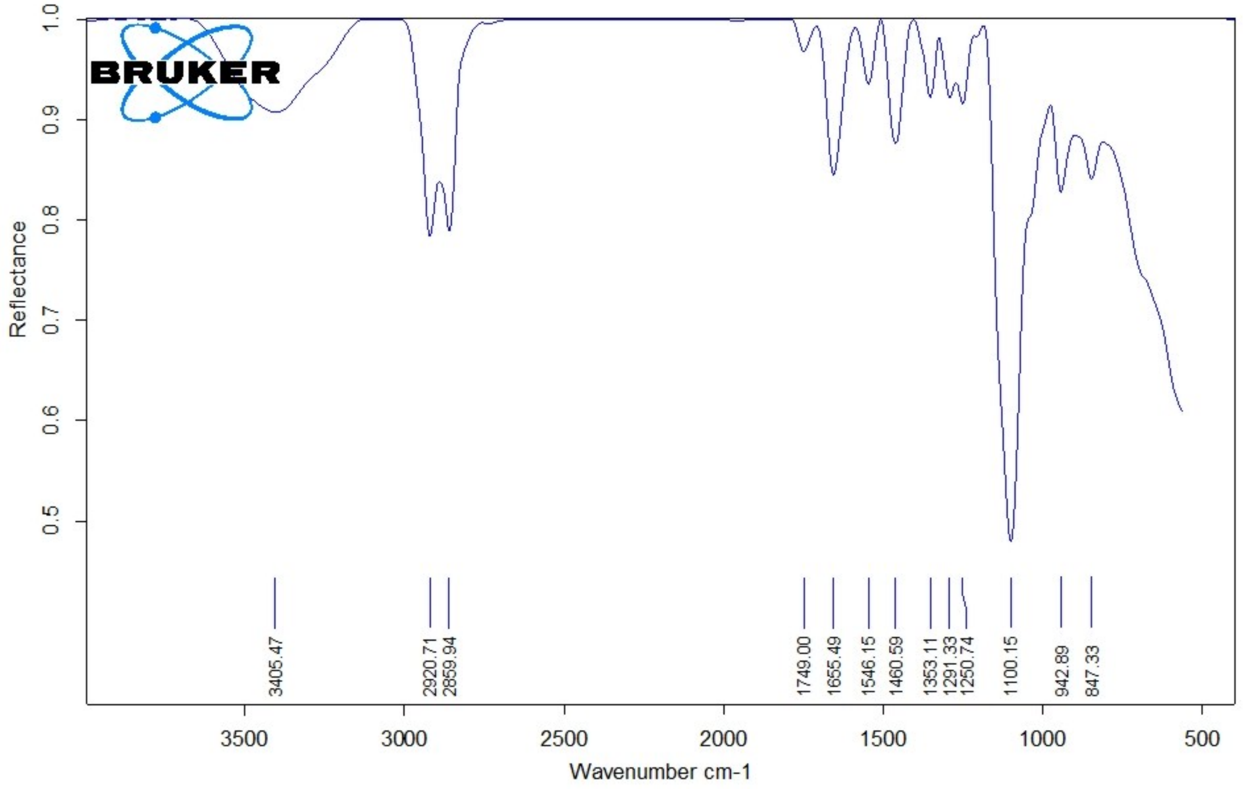

Fig. S3. FT-IR spectrum of cationic poly(ethylene oxide) gemini surfactant (**GS3**)

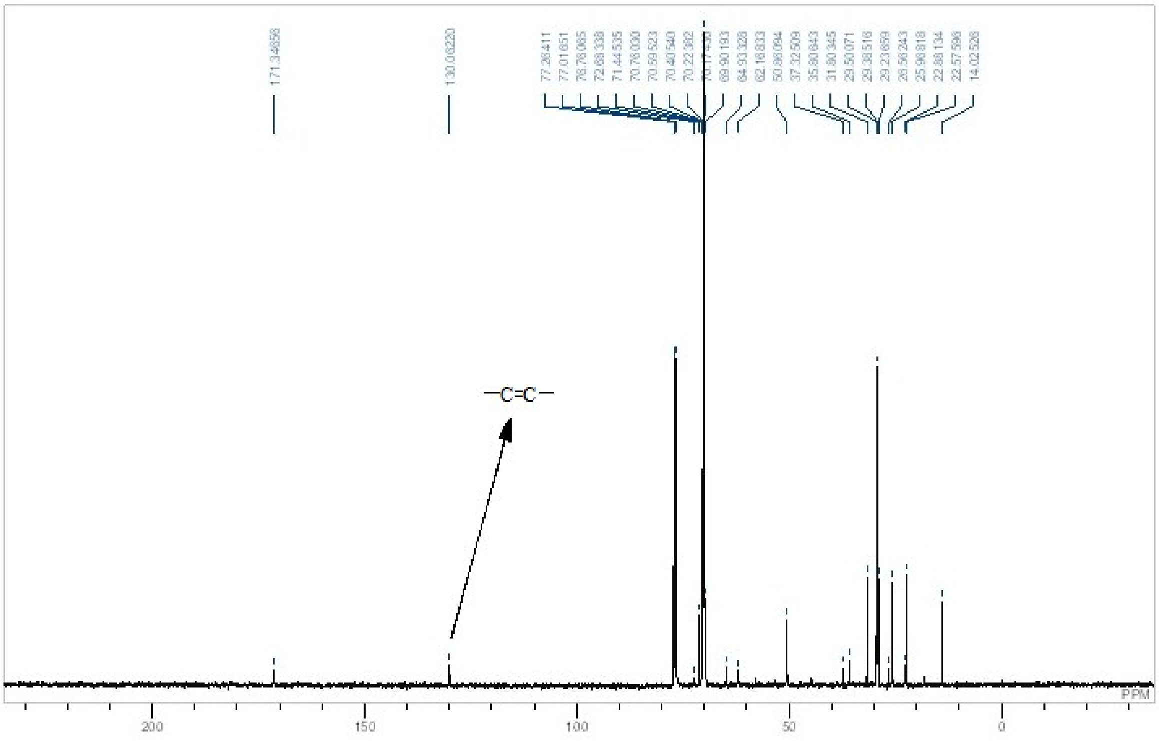

Fig. S4. ^{13}C-NMR of cationic poly(ethylene oxide) gemini surfactant (**GS2**)

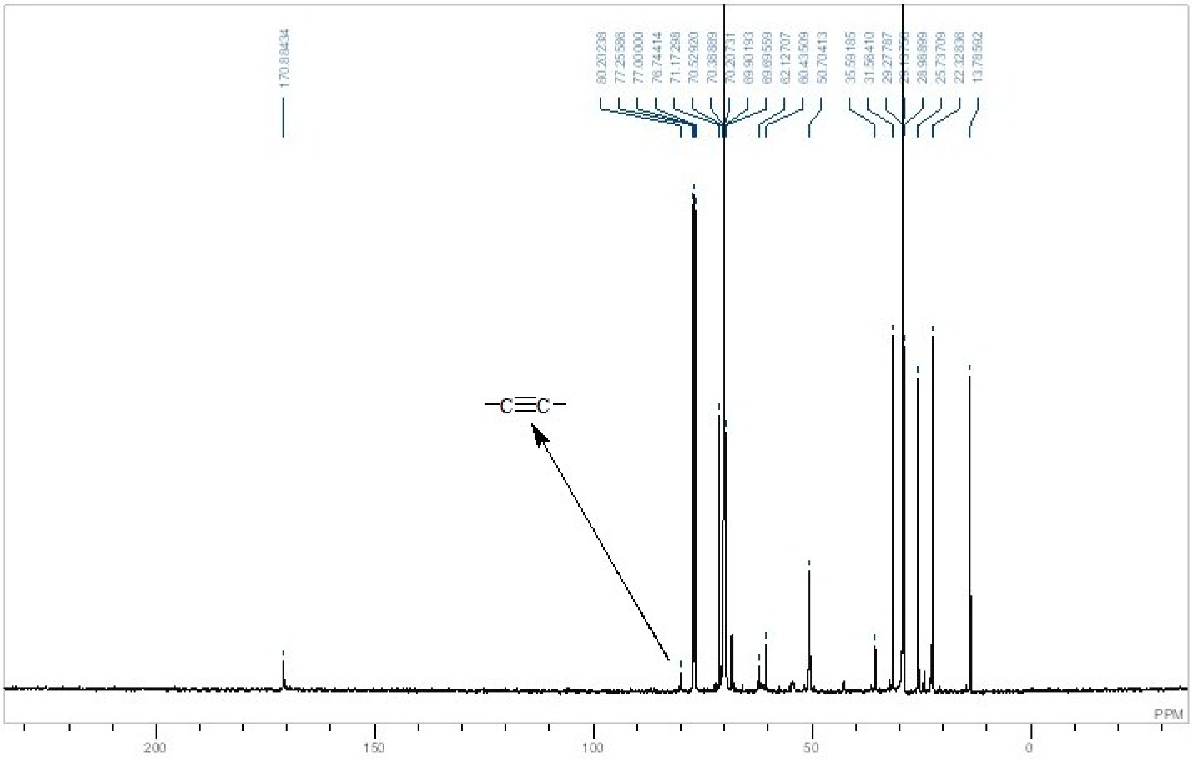

Fig. S5. ^{13}C-NMR of cationic poly(ethylene oxide) gemini surfactant (**GS3**)

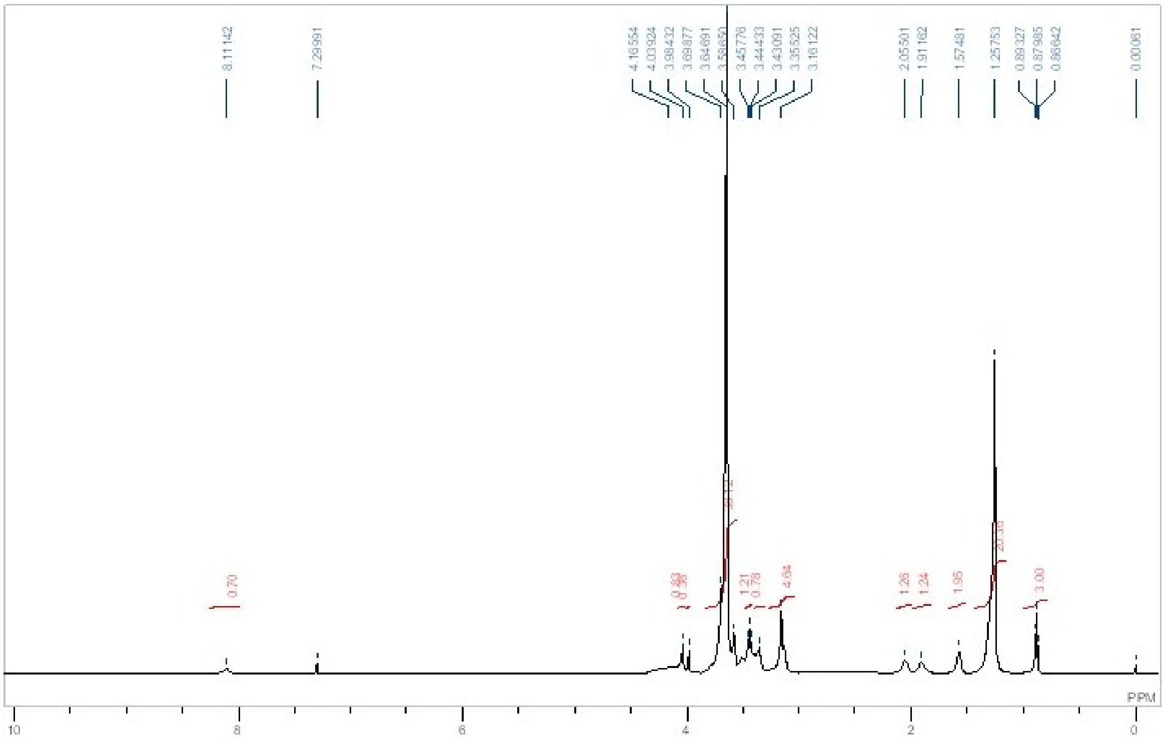

Fig. S6. ^{1}H-NMR of cationic poly(ethylene oxide) gemini surfactant (**GS1**)
